# Highly Sensitive, Calibration-Free WM-DAS Method for Recovering Absorbance—Part I: Theoretical Analysis

**DOI:** 10.3390/s20030681

**Published:** 2020-01-26

**Authors:** Zhimin Peng, Yanjun Du, Yanjun Ding

**Affiliations:** State Key Laboratory of Power Systems, Department of Energy and Power Engineering, Tsinghua University, Beijing 100084, China; apspect@tsinghua.edu.cn (Z.P.);

**Keywords:** tunable diode laser absorption spectroscopy, absorbance, fast-Fourier transform, calibration-free, gas temperature and concentration, spectroscopic parameters

## Abstract

The absorbance is of great importance in the tunable diode laser absorption spectroscopy (TDLAS) as it contains information of both gas properties and spectroscopic parameters. A novel, calibration-free wavelength modulation-direct absorption spectroscopy (WM-DAS) is proposed and experimentally verified in this two-part paper. This method combines the capability of absorbance measurement from DAS and the advantages of enhanced noise rejection and high sensitivity from WMS. In this *Part I*, we focus on the full theoretical basis and procedures of this method from the following three aspects: the high-accuracy characterizations of laser frequency and intensity, noise rejection ability by extracting the characteristic spectra through the fast Fourier transform (FFT) of the light intensity, and the simultaneous fitting strategy for both baseline and absorbance. The preliminary validation experiment of CO transition at 4300.6999 cm^−1^ in a static gas cell shows the high accuracy of the proposed method.

## 1. Introduction

In the past several decades, the tunable diode laser absorption spectroscopy (TDLAS) has played a growing role in the diagnostic of combustion and plasmas, as well as in some industrial applications [1,2,3,4,5,6,7,8,9]. As the key parameter in TDLAS, the absorbance contains the information of both gas properties (temperature, pressure, concentration, velocity, etc.) and the spectroscopic parameters (line strength, collisional broadening, narrowing coefficients, etc.). Therefore, much attention has been paid to the accurate determination of the absorbance [10,11,12,13]. 

Typically, absorbance is acquired through a triangular or sawtooth wave-scanned direct absorption spectroscopy (DAS), which is easy to implement and has relatively straightforward data processing [13,14,15]. However, this method suffers from these following problems in practice: first, the uncertainty in the baseline determination reduces the accuracy of the absorbance. In most conventional approaches of DAS, the baseline (without absorption) is usually fitted with a polynomial using the non-absorption portions in the time domain, upon which the absorbance profile can be obtained based on the Beer-Lambert law and laser wavelength measurement. Nevertheless, the extraction of the real non-absorption portions is quite challenging because of the line overlapping and the limited scan range of typical diode lasers [9]. As estimated in Ref. [16], a 1% relative error in the baseline fitting for absorption of 1% results in a 100% error in the peak measurement in DAS. Second, the ramp waveform that is intended to ensure a relatively simple baseline form has a large requirement on the bandwidth of the detection system. Thus, the scan frequency is typically limited within the range from 100 Hz to 10 kHz [17], which ultimately restraints the time resolution of DAS and renders it susceptible to low-frequency noises such as vibrations, particles, laser 1/*f*, etc. In addition, the sudden change in the laser frequency (or its derivative) caused by the sudden change of current at the peak or valley of the sawtooth (triangular) wave brings additional difficulties to the laser wavelength characterization near these turning points.

Many efforts have been made to overcome these issues in DAS. For the baseline fitting issue, both the time-division and beam-splitting strategies were introduced attempting to directly measure the baseline instead of fitting with the non-absorption portions [13,14,18]. However, the time-division strategy, where the baseline is measured before (or after) the experiment with the optical path purged, only applies to certain circumstances, such as spectroscopic parameters measurement in a chamber, but not to those with exposed absorption area, i.e., combustion diagnostic and practical field sensing. The beam-splitting method, where the baseline is measured with a split laser going through a reference cell, works well with the NIR laser, while it is not practical for the MIR range because of the immature, expensive, and complicated laser splitting system. Moreover, the time-division strategy is susceptible to the temporal laser fluctuation and light path distortion, and the beam-splitting method is easily affected by the potential errors from additional optical etalons. Some other efforts had also been made for the baseline fitting issue by using a sinusoidal wavelength scan that provides an explicit baseline form in the time domain [19]. As for the temporal resolution of DAS, although quick response can be achieved by measuring transmitted intensities at a fixed center frequency, the influences from vibration, optical path shift, and radiation on transmitted intensity are still unable to eliminate.

In contrast to DAS, wavelength modulation spectroscopy (WMS) using harmonic detection technology can effectively eliminate the influence of baseline uncertainty and work with much higher modulation frequency (as high as several hundred kHz) [20,21,22,23]. Therefore, WMS is resistant to the low-frequency noise and has a higher sensitivity. Since introduced, WMS technology has been widely studied and used for the gas properties measurement [24,25,26,27]. In the early stages, the measurement strategy based on the 2*f* peak value and the calibration with a known gas mixture is widely used. Later, several researchers, including Hanson et al. proposed calibration-free WMS techniques [28,29,30,31] that present a distinct advantage in the practical application. Although WMS has already been widely used on the diagnostic of gas properties, few studies have been reported on the measurement of absorbance and further the spectroscopic parameters using WMS. The main reasons are the multi-parameter coupling and the complexity of harmonic signals. Specifically, the harmonics not only include the absorption information (which depends on gas temperature, pressure, concentration, optical length, spectroscopic constants, and so on), but also affected by the laser performance (including light intensity, the phase difference between intensity and frequency modulation, the modulation index of frequency, etc.) and response of the detecting system. The uncertainties from all these components deviate the theoretical or simulated harmonics from their actual values, and thus, arise errors in the measurement.

Considering the importance of the absorbance and the high sensitivity of the harmonic, recently, attempts have been made to recover the absorbance using the harmonic signals [10,11,32,33,34,35,36]. G. Stewart [10,11,32] proposed a method to recover the absorbance shape using the first harmonic signal based on RAM (residual amplitude modulation). This method works well under small modulation indices (*m* < 0.2) when the *X* or *Y* component of the first harmonic is close to the absorbance shape. However, the higher-order terms of the modulation index strongly affect the recovered results when greater modulation indices are used, therefore the recovering errors increase sharply with the increasing modulation index. In our research [34], we proposed a method that employs additional higher odd harmonics (3rd, 5th…) to enhance the recovering accuracy with large modulation indices. By using all the odd harmonics, the contributions of the modulation index’s higher-order terms to the recovering error are eliminated. Results from the simulation and experiment show that this method works well on the absorbance profile recovery regardless of the modulation index. However, the higher odd harmonics always come with the low signal-to-noise ratio (SNR) and are difficult to obtain in actual measurements.

Inspired by the above-mentioned works, in reference [37], we first proposed the preliminary idea of the WM-DAS method, which directly measures the absorbance by extracting the characteristic frequencies of the modulated light intensity based on the FFT analysis. However, the accuracy of the laser light intensity and frequency model in [37] is still limited. Meanwhile, the baseline in Ref. [37] has to be experimentally measured, which is not practical in some conditions. In this paper, we further developed the WM-DAS method from the following two aspects. First, more accurate models for laser intensity and wavelength are developed with full consideration of the nonlinearity effect. Second, a simultaneous fitting algorithm was developed to deduce both baseline and the absorbance based on the accurate model of baseline. Therefore, although the preliminary idea of this method has been reported from our group [37], this paper is still significant for generalizing and developing it toward a fully-fledged method. To provide a better understanding and an easy implementation of the WM-DAS method to readers, several crucial equations that have been reported in reference [37] are also included in this manuscript.

To clearly explain, validate, and promote the application of this method, the whole work is presented in two parts, *Part I*—*Theoretical Analysis* and *Part II*—*Experimental Investigation*. In *Part I*, the WM-DAS method was established through theoretical derivation and preliminarily validated with the CO (2-0) *R*(11) absorption line at 4300.6999 cm^−1^ in a static cell. The features of the proposed method and its potential application are demonstrated using experimental results and discussed in *Part II*.

*Part I*—Theoretical analysis has the following objectives and contents:

- **Present the theoretical derivation of the proposed WM-DAS method.** First, the non-linear laser frequency response with the high-frequency sinewave modulation is optimized by considering the 3*ω* components. Then, by introducing the intermediate variable *x*, the relationship between the light intensity and frequency can be established through the FFT analysis and the extraction of the characteristic frequencies of the light intensity.
- **Develop the simultaneous fitting** algorithm **for the recovery of absorbance.** By establishing the relationship between the baseline and variable *x* based on the sinusoidal current scan, a fitting algorithm is developed to simultaneously infer the baseline and the more important absorbance.
- **Summarize the procedure of the proposed WM-DAS method.**

*Part II*—Experimental investigation mainly focuses on the following contents:

- **Experimentally validate the main properties of the proposed WM-DAS method.** The ability of noise rejection, temporal resolution, and the performance with small scan indexes are checked in a high-temperature tube furnace taking the CO transition at 4300.6999 cm^−1^ as an example.
- **Demonstrate the real experimental applications of the proposed WM-DAS method.** Both the high-accuracy measurement of the spectroscopic parameters and the diagnostic of a flat flame on McKenna burner are presented. The collisional broadening and Dicke narrowing coefficient and their corresponding temperature exponents of the CO transition are measured. Further, the CO concentration and temperature of a standard flat flame at different height and stoichiometric ratios are discussed.

## 2. Materials and Methods

**Figure 1:**
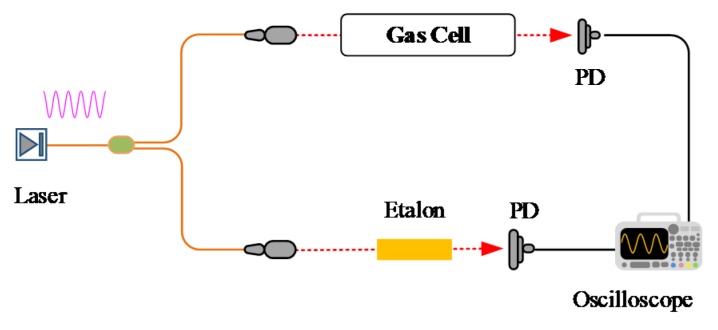
Experimental setup used for the validation of the proposed wavelength modulation-direct absorption spectroscopy (WM-DAS) method.

In contrast to the triangular (or sawtooth) wave in DAS or the triangular wave superimposed by a high-frequency sine wave in WMS, pure sinusoidal signal with frequency *ω* is used to scan around the absorption line in WM-DAS. As shown in Figure 1, the instantaneous modulation frequency of a typical distributed-feedback (DFB) laser can be written as follows,

(1) $$\begin{array}{cc} v\left(t\right)=\bar{v}+\sum_{j=1}^{n}a_{j}\cos\left[j\cdot \left(\omega t+\eta \right)+\varphi_{j}\right] & j=1,2\cdots , \end{array}$$

where *η* is the initial phase of the base frequency, $\bar{v}$ is the laser center wavelength. *a_j_* and *φ_i_* are the modulation depth and phase shift of the *i*^th^-order frequency modulation (FM), and *φ*_1_ = 0. Similarly, instantaneously modulated incident intensity can be written as,

(2) $$\begin{array}{cc} I_{0}=\bar{I}+\sum_{k=1}^{\infty }i_{k}\cos\left[k\left(\omega t+\eta \right)+\theta_{k}\right] & \;k=1,2\cdots , \end{array}$$

where $\bar{I}$ is the DC laser intensity. *i_k_* and *θ_k_* are the modulation amplitudes and phase shift of the *i*^th^-order intensity modulation (IM).

To obtain the absorbance, the relationship between the laser wavelength (Equation (1)) and intensity (Equation (2)) is essential in WM-DAS. Here, we define variable *x* as [37]

(3) x=cos(ωt+η) x∈[−1,1].

### 2.1. Relationship between the Laser Wavelength and Variable x

Considering the large scan range of the laser wavelength and the laser’s nonlinear response to the injected current tuning, high order terms (2*ω* and 3*ω*) are used to describe the instantaneous laser frequency in WM-DAS.

(4) $$v\left(t\right)=\bar{v}+a_{1}\cos\left(\omega t+\eta \right)+a_{2}\cos\left[2\left(\omega t+\eta \right)+\varphi_{2}\right]+a_{3}\cos\left[3\left(\omega t+\eta \right)+\varphi_{3}\right].$$

**Figure 2:**
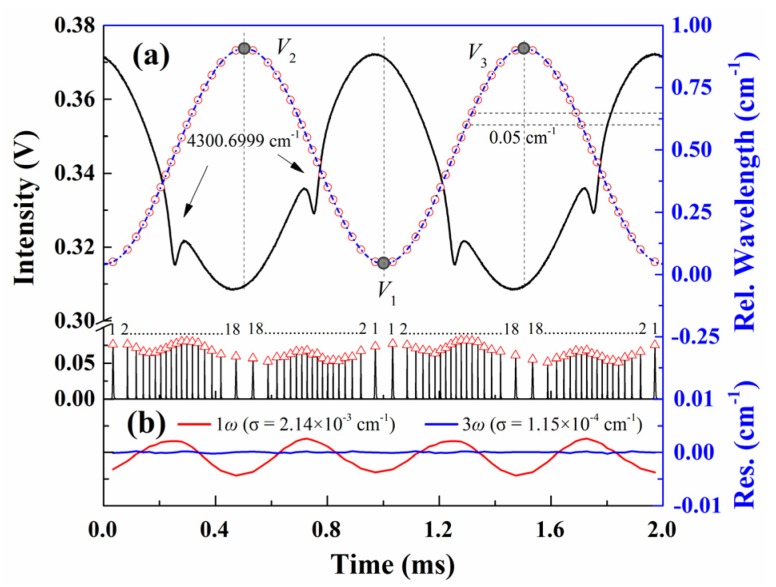
(**a**) Detected transmitted light intensity and etalon fringe peak results together with the best fit of the relative wavelength. (**b**) Fitting residuals of the best-fit relative wavelength. The experimental conditions are: CO/N_2_ mixture gas, *T* = 299 K, *L* = 14.7 cm, *Xco* = 1.02%, *P* = 101.3 kPa.

By substituting Equation (3) into Equation (4), the relationship between *v* and variable *x* can be written as follows,

(5) $$\begin{array}{c} v=\bar{v}+a_{1}x+a_{2}\cdot \left[\left(2x^{2}-1\right)\cos\varphi_{2}\right]\pm 2x\sin\varphi_{2}\sqrt{1-x^{2}}\\ +a_{3}\cdot \left[\left(4x^{3}-3x\right)\cos\varphi_{3}\pm \left(4x^{2}-1\right)\sqrt{1-x^{2}}\sin\varphi_{3}\right] \end{array},$$

where the symbols “-” and “+” apply to the wavelength behavior in the *V*_1_*V*_2_ (left) and *V*_1_*V*_3_ (right) periods, respectively, as shown by the blue dashed curve in Figure 2a. If the non-linear effect in Equation (4) is ignored (*a*_2_ = *a*_3_ = 0), the laser wavelength responses in both the *V*_1_*V*_2_ and *V*_1_*V*_3_ periods are symmetric and proportional to the variable *x*.

(6) $$v=\bar{v}+a_{1}x.$$

To test the accuracy of Equations (4) and (5) in the description of the laser frequency response, an experimental study on CO (2←0) R (11) transition at 4300.6999 cm^−1^ was conducted according to the setup in Figure 1. A sine wave with a frequency of *f* = 1 kHz and an amplitude of ±10 mA@140 mA was used to modulate the DFB laser (NORCADA Canada). The relative frequency of the DFB laser was characterized with a Fabry–Perot etalon (Thorlabs SA200-18C) with a free spectral range (FSR) of 1.5 GHz ± 3 MHz. The nominal FSR value was experimentally checked by measuring the spectral interval between two strong CO transitions (4300.699869 cm^−1^ and 4303.623335 cm^−1^ tabulated in HITRAN 2016 [38]). Although the measured FSR is 1.498 GHz, which agrees well within the uncertainty with the nominal value, the nominal FSR, 1.5 GHz, was used in the manuscript considering the uncertainty in the line positions. Signals from the FP etalon and the transmitted light intensity detected by the HgCdTe detector (Vigo PVI-2TE-3) are shown in Figure 2. The detected etalon fringe peaks are marked with red “Δ” symbols, and the wavelength interval between two adjacent peaks equals the FSR (0.05 cm^−1^). The reassigned relative wavelength points are shown by the pink “○” symbols together with its best-fit curve with Equation (4). As illustrated in Figure 2b, the best-fit residual that considers only the linear response, marked by the red line, presents pronounced sinusoidal structure at twice the modulation frequency, indicating the non-ignorable 2*ω* nonlinear term [37]. In contrast, the best-fit residual with Equation (4), shown by the blue line, is reduced by more than one order of magnitude with a standard deviation as small as 1.15 × 10^−4^ cm^−1^. The best-fit parameters $\bar{v}$, *a*_1_, *a*_2_, *a*_3_, *η*, *φ*_2_, and *φ*_3_ in Equation (4) are 4.777 × 10^−1^ cm^−1^, 4.332 × 10^−1^ cm^−1^, 3.193 × 10^−3^ cm^−1^, 2.181 × 10^−4^ cm^−1^, 0.9937 π, −0.9169 π, and −0.9868 π, respectively.

**Figure 3:**
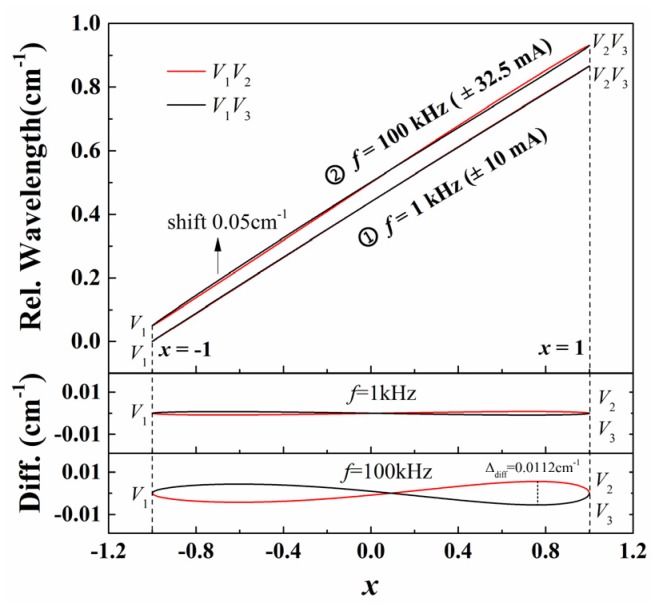
The relationship between laser wavelength and variable *x* in both *V*_1_*V*_3_ and *V*_1_*V*_2_ edges for different modulation frequencies 1 kHz and 100 kHz.

The laser frequency as a function of variable *x* in the *V*_1_*V*_2_ and *V*_1_*V*_3_ edges can be obtained by substituting the above best-fitted parameters, $\bar{v}$, *a*_1_, *a*_2_ and so on, into Equation (5), as shown by the lines of group ① in Figure 3. The bottom panels show how much the laser frequency deviates from its average, which has a clear ∞-shaped structure and equals 0 at two ends (*x* = ±1). As shown in Equation (6), when the nonlinearity is ignored (*a*_2_ = *a*_3_ = 0), the laser wavelength curves in both edges merge into one line. Additionally, this discrepancy between both edges becomes more prominent as the modulation frequency increases for the same laser. Lines in group ② in Figure 3 illustrate the laser wavelength response with a modulation frequency *f* = 100 kHz (modulation current ± 32.5 mA@140 mA, wavelength scan range *a* = 0.881 cm^−1^, current coefficient 0.0136 cm^−1^/mA). The largest difference between the two edges reaches 0.0112 cm^−1^. Therefore, considering the significant nonlinearity of laser frequency response, an accurate relationship between laser frequency and *x*, as described by Equation (5), is critical to the precise recovery of absorbance in WM-DAS.

### 2.2. Relationship between the Laser Intensity and Variable x

Based on the Beer–Lambert law, the transmitted light intensity of a monochromatic laser at frequency *ν* through a uniform medium can be expanded as follows [37]:

(7) $$I_{t}=I_{0}\cdot \exp[-\alpha (v)]=\sum_{k=0}^{\infty }X_{k}\cdot \cos\left(k\omega t\right)-\sum_{k=0}^{\infty }Y_{k}\cdot \sin\left(k\omega t\right)\;k=0,1,2\ldots ,$$

where *I*_0_ and *α*(*v*) are the incident light intensity and the absorbance. *X_k_* and *Y_k_* are the *k*^th^ Fourier coefficients of the transmitted intensity. *ω* is the angular frequency of the current modulation.

As defined in Equation (3), variable *ωt* in *V*_1_*V*_2_ and *V*_1_*V*_3_ edges can be written as follows,

(8) $$\omega t=\left\{ \begin{array}{l} 2n\pi +\arccos x-\eta ,\;n=0,1,2\ldots \;(V_{1}V_{2})\\ 2n\pi -\arccos x-\eta ,\;n=1,2,3\ldots \;(V_{1}V_{3}) \end{array}\right..$$

By substituting Equation (8) into Equation (7), the relationship between laser intensity and variable *x* can be obtained as,

(9) $$I\left(x\right)=\sum_{k=0}^{\infty }X_{k}\cdot \cos\left[k\cdot \left(\arccos x\pm \eta \right)\right]\pm \sum_{k=0}^{\infty }Y_{k}\cdot \sin\left[k\cdot \left(\arccos x\pm \eta \right)\right],$$

where the symbols “−” and “+” apply to the laser intensity correspond to the *V*_1_*V*_2_ and *V*_1_*V*_3_ periods, respectively.

### 2.3. Relationship between Laser Intensity and Wavelength

**Figure 4:**
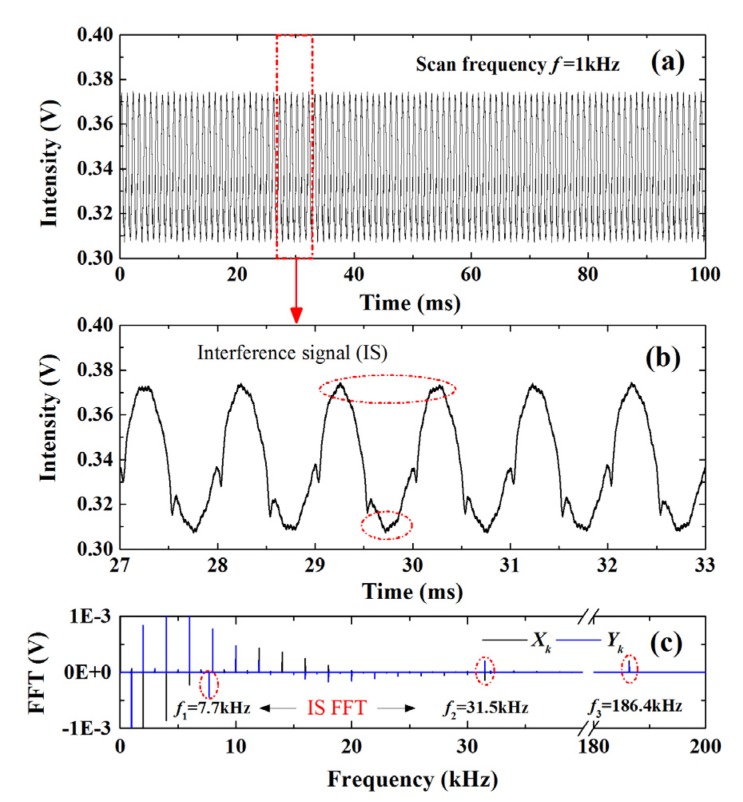
(**a**) Detected transmitted light intensity with several disturbance signals. (**b**) The enlarged figure of the transmitted light intensity. (**c**) Fourier coefficients *X_k_* and *Y_k_* of the transmitted light intensity with 100 cycles. The experimental conditions are: CO/N_2_ mixture gas, *T* = 299 K, *L* = 14.7 cm, *Xco* = 1.02%, *P* = 101.3 kPa.

As discussed in Section 2.1 and Section 2.2, the laser wavelength and intensity in WM-DAS are expressed as a function of the intermediate variable *x* with high accuracy. Thus, light intensity and laser wavelength can be directly correlated by neutralizing variable *x* in Equations (5) and (9). In this work, an experimental study on the CO spectral line at 4300.6999 cm^−1^ was conducted using the experimental system shown in Figure 1. The ultimate vacuum of the gas cell was 10^−4^ Pa, with a leakage rate of 0.02 Pa/min. During the experiment, the gas cell was pumped to 10^−2^ Pa and then filled with 1.02% CO/N_2_ mixture to 101.3 ± 0.01 kPa. The effective optical length in the cell is 14.7 cm and the entire experiment was conducted at room temperature (299 ± 0.1 K). The laser scan rate, modulation amplitude, and the wavelength scan range are 1 kHz, ± 10 mA, and 0.866 cm^−1^, respectively. The laser wavelength response is shown by the line group ① in Figure 3 and the transmitted light intensity is shown in Figure 4.

As discussed in Section 2.1 and Section 2.2, the laser wavelength and intensity in WM-DAS are expressed as a function of the intermediate variable *x* with high accuracy. Thus, light intensity and laser wavelength can be directly correlated by neutralizing variable *x* in Equations (5) and (9). In this work, an experimental study on the CO spectral line at 4300.6999 cm^−1^ was conducted using the experimental system shown in Figure 1. The ultimate vacuum of the gas cell was 10^−4^ Pa, with a leakage rate of 0.02 Pa/min. During the experiment, the gas cell was pumped to 10^−2^ Pa and then filled with 1.02% CO/N_2_ mixture to 101.3 ± 0.01 kPa. The effective optical length in the cell is 14.7 cm and the entire experiment was conducted at room temperature (299 ± 0.1 K). The laser scan rate, modulation amplitude, and the wavelength scan range are 1 kHz, ± 10 mA, and 0.866 cm^−1^, respectively. The laser wavelength response is shown by the line group ① in Figure 3 and the transmitted light intensity is shown in Figure 4.

To further investigate the noise rejection ability of the proposed WM-DAS method, artificial noise signals at fixed frequencies (7.7 kHz, 31.5 kHz, and 186.4 kHz) were injected into the experimentally recorded transmitted intensity signal. Figure 4a plots the transmitted light intensity with noises (100 periods with 1000 points per period), and the section encircled by the red dashed line is enlarged in Figure 4b to highlight the detailed features. Clear distortions can be observed especially at the peak and valley locations. Figure 4c shows the Fourier coefficients of the detected transmitted light signal in Figure 4a. As can be seen, the frequency spectrum containing the information of absorption mainly locates at the integer multiples of the base frequency *kf* (*k* = 0, 1, 2…) and the FFT coefficient decrease rapidly with the increasing frequency order. Meanwhile, as labeled by the red dashed circles in Figure 4c, the frequency spectrum of the noise signals is pronounced at frequencies of 7.7 kHz, 31.5 kHz, and 186.4 kHz.

**Figure 5:**
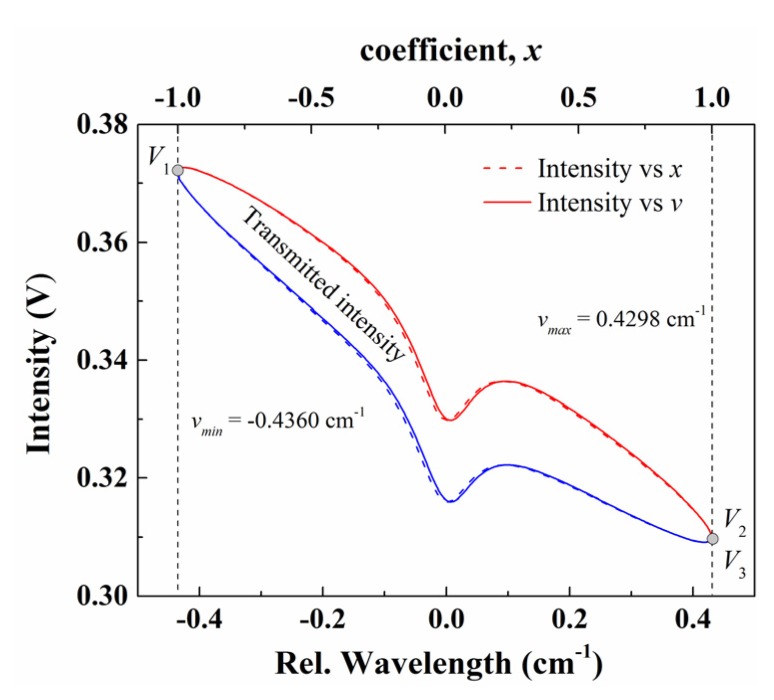
The recovered relationship between the transmitted light intensity and laser wavelength in WM-DAS. The experimental conditions are: CO/N_2_ mixture gas, *T* = 299 K, *L* = 14.7 cm, *Xco* = 1.02%, *P* = 101.3 kPa.

By substituting these FFT coefficients at *kf* (*k* = 0, 1, 2,…) frequencies into Equation (9), the relationship between the transmitted light intensity and *x* in *V*_1_*V*_2_ and *V*_1_*V*_3_ sections are obtained as shown by the red and blue dash lines in Figure 5, respectively. Furthermore, through the *x*→*v* (frequency) transformation in Equation (5), the relationship between laser intensity and wavelength can be obtained, as shown by the solid lines in Figure 5. Clearly, the nonlinear response of the laser wavelength leads to the discrepancy between solid and dash lines, and this discrepancy becomes more significant as the modulation frequency increases. It is also worth noticing that the distortions caused by the noises in Figure 4 vanish in the recovered light intensity in Figure 5, which supports the fact that the random noise and noises with other frequencies can be effectively eliminated in WM-DAS method, as only the FFT coefficients at *kf* (*k* = 0, 1, 2 …) frequency are used in the purposed method.

## 3. Recovery of the Absorbance

Analogous to DAS, a precise determination of the baseline is essential to measure the absorbance in WM-DAS.

### 3.1. Description of the Baseline Based on a Sinusoidal Modulation

As shown in Figure 5, the baseline cannot be simply described by a polynomial. Therefore, in this section, we derived the expression of the original baseline based on the sinusoidal modulation signal, which is further used for high-precision recovery of absorbance. Similar to the wavelength description, we also consider the nonlinearity effect in the laser intensity response as follows,

(10) $$I_{0}=\bar{I}+i_{1}\cos\left[\left(\omega t+\eta \right)+\theta_{1}\right]+i_{2}\cos\left[2\left(\omega t+\eta \right)+\theta_{2}\right]+i_{3}\cos\left[3\left(\omega t+\eta \right)+\theta_{3}\right],$$

where $\bar{I}$ is the DC laser intensity. *i*_1_, *i*_2_, and *i*_3_ are the linear and nonlinear intensity modulation (IM) amplitudes, and *θ*_1_, *θ*_2_, and *θ*_3_ are the linear and nonlinear IM phase difference between the IM and the FM, respectively. By substituting Equation (3) into Equation (10), the baseline can be described by variable *x* as follows,

(11) $$\begin{array}{c} I_{0}=\bar{I}+i_{1}\left[x\cos\theta_{1}\pm \sqrt{1-x^{2}}\sin\theta_{1}\right]+i_{2}\cdot \left[\left(2x^{2}-1\right)\cos\theta_{2}\right]\pm 2x\sqrt{1-x^{2}}\sin\theta_{2}\\ +i_{3}\cdot \left[\left(4x^{3}-3x\right)\cos\theta_{3}\pm \left(4x^{2}-1\right)\sqrt{1-x^{2}}\sin\theta_{3}\right] \end{array}.$$

For simplification, we define the following parameters,

(12) $$\begin{array}{llll} B_{0}=\bar{I}-i_{2}\cos\theta_{2}, & B_{1}=i_{1}\cos\theta_{1}-3i_{3}\cos\theta_{3}, & B_{2}=2i_{2}\cos\theta_{2},\\ B_{3}=4i_{3}\cos\theta_{3}, & B_{4}=i_{1}\sin\theta_{1}-i_{3}\sin\theta_{3}, & B_{5}=2i_{2}\sin\theta_{2}, & B_{6}=4i_{3}\sin\theta_{3}. \end{array}$$

Thus, the baseline expression is:

(13) $$I_{0}=B_{0}+B_{1}x+B_{2}x^{2}B_{3}x^{3}\pm (B_{4}+B_{5}x+B_{6}x^{2})\sqrt{1-x^{2}}.$$

Similar to the analysis for laser wavelength, when the non-linearity of laser intensity is neglected (*i*_2_ = *i*_3_ = 0), we have:

(14) $$I_{0}=\bar{I}+i_{1}\left[x\cos\theta_{1}\pm \sqrt{1-x^{2}}\sin\theta_{1}\right].$$

The “-” and “+” symbols denote the incident intensities in *V*_1_*V*_2_ and *V*_1_*V*_3_ frequency range, respectively, in Equations (11), (13), and (14). It must be mentioned that according to Equation (14), even if the non-linearity of laser intensity is neglected, the intensity in *V*_1_*V*_2_ and *V*_1_*V*_3_ range is not proportional to *x*. This is caused by the phase difference *θ*_1_ between FM and IM. When the phase difference *θ*_1_ equals π, Equation (14) becomes a linear formula. However, in practical measurement, the phase difference *θ*_1_ varies in (π, 1.5π), and increases with the modulation frequency, as measured in reference [21].

**Figure 6:**
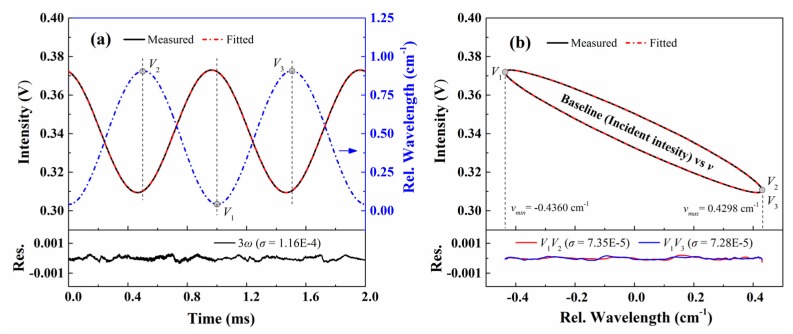
(**a**) Detected baseline versus time and its best-fit with Equation (10); (**b**) recovered baseline versus wavelength form WM-DAS and its best-fit with Equation (13). The experimental conditions are: pure N_2_, *T* = 299 K, *L* = 14.7 cm, *P* = 101.3 kPa.

To examine the accuracy of Equation (10) on describing the instantaneous laser intensity (without absorption), we measured the incident light intensity by pumping the sample cell to 10^−2^ Pa, then filling the cell with pure N_2_ to 101.3 kPa. The laser modulation rate (*f* = 1 kHz) and wavelength scan range (0.866 cm^−1^) are identical to the previous experiments. The detected baseline intensity (averaged by 100 periods), and its best-fit curve with Equation (10) are presented in Figure 6a. The relative fitting residual normalized by the absolute light intensity is also attached to the bottom panel. The standard deviation of the normalized fitting residual is only 1.16 × 10^−4^, showing that the description of the temporal laser intensity using Equation (10) is of high accuracy.

Meanwhile, the relationship between baseline and laser wavelength recovered by the proposed WM-DAS method is shown in Figure 6b. The curve shows an elliptic shape with apparent blending near the minimum frequency (*V*_1_) and the maximum frequency (*V*_2_ and *V*_3_), and such a bending feature becomes more noticeable when the scan rate increases. Apparently, the polynomial fitting used in DAS is incapable to describe this relationship precisely. In contrast, the best fits of the detected baseline with Equation (13) for both *V*_1_*V*_2_ and *V*_1_*V*_3_ edges are shown by the red dash-dot line, together with their residuals below. The standard deviations of the normalized fitting relative residual are 7.35 × 10^−5^ and 7.28 × 10^−5^ for *V*_1_*V*_2_ and *V*_1_*V*_3_ sections, respectively, which are much less than those in Figure 6a. This also indicates that the proposed method is immune to noise.

### 3.2. Simultaneous Recovery of Both Absorbance and Baseline

To avoid the uncertainty caused by the independent baseline fitting, the baseline and absorbance are simultaneously recovered in WM-DAS using the baseline expression in Section 3.1 and Beer–Lambert law:

(15) $$I_{v}=I_{0}\cdot \exp[-\alpha (v)],$$

where *I_v_* is the transmitted intensity recovered using WM-DAS method, *I*_0_ is the baseline fit by Equation (13), both of which are expressed as functions of wavelength. As for the absorbance *α*(*v*), here we adopt the Rautian line profile, which takes the Dicke narrowing effect into consideration [37]. From Equation (15) we know that both the absorbance and baseline can be directly deduced by the least-square fitting of the recovered transmitted light intensity from Section 2 with the baseline formula in Section 3.1.

**Figure 7:**
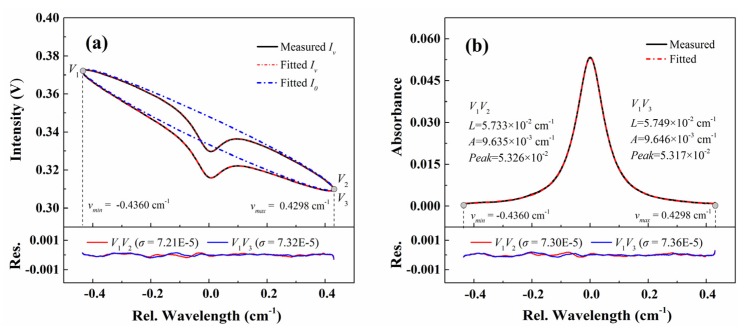
(**a**) Best-fit transmitted light intensity and baseline from the proposed WM-DAS. (**b**) The measured absorbance calculated with the best-fit baseline. The experimental conditions are: CO/N_2_ mixture gas, *T* = 299 K, *L* = 14.7 cm, *Xco* = 1.02%, *P* = 101.3 kPa.

To validate the accuracy of this simultaneous recovery process, we applied the proposed method to analyze the transmitted light intensity in Figure 5. In the fitting program, the Gauss line width is fixed at 5.03 × 10^−3^ cm^−1^, calculated with the experimental temperature 299 K. The best-fit transmitted light intensity and the baseline are shown in Figure 7a together with the relative residual in below. The residuals and standard deviations for both edges are similar to those in Figure 6b, which indicates that the absorption feature did not introduce additional fitting residuals, and thus, suggesting an accurate absorbance determination. Furthermore, based on the recovered transmitted light intensity and the best-fit baseline in Figure 7a, the absorbance can be calculated as shown in Figure 7b. As can been seen, even with a relatively small absorption (peak absorption of 5.3%) and large line broadening (at atmospheric pressure), the standard deviation of the best-fit residual remains as small as 7.3 × 10^−5^, which endows the proposed WM-DAS method with high SNR and low detection limit. The related discussion will continue in *Part II*.

Moreover, as listed in Figure 7b, the collisional broadenings, integrated area and peak absorptions inferred from *V*_1_*V*_2_ and *V*_1_*V*_3_ edges are consistent with relative differences smaller than 0.3%. This also suggests the high precision and reproducibility of the proposed WM-DAS method. The collisional broadening and integrated area averaged for both sides are used in the following discussion. The inferred gas concentration calculated from the average integrated area (9.641 × 10^−3^ cm^−1^), pressure (101.3 kPa), line strength (6.410 × 10^−2^ cm^−1^/atm @ 299 K from HITRAN 2016 [38]) and optical length (14.7 cm) is 1.023%, which has only 0.3% difference with the nominal value 1.02%. Besides, the measured collisional broadening (5.741 × 10^−2^ cm^−1^/atm, *T* = 299 K) is slightly larger than the data from HITRAN 2016 (5.604 × 10^−2^ cm^−1^/atm @ 299 K). This may result from the utilization of the Voigt profile in Ref. [39], which ignores the effects of collisional and speed-dependent narrowing and finally yields an underestimation of the collisional width [19].

## 4. Overview of the WM-DAS Method

**Figure 8:**
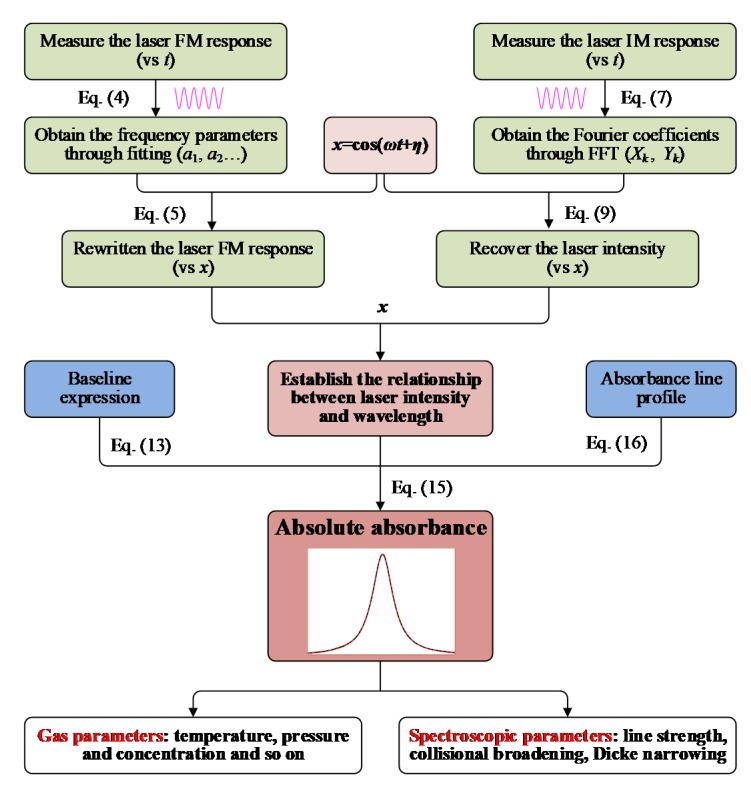
Flowchart for the proposed WM-DAS method.

The flowchart of the proposed WM-DAS method is presented in Figure 8. First, the laser wavelength in the time domain is measured with an interferometer, through which the corresponding parameters including *a*_1_, *a*_2_, *φ*_2_ can be fitted. Next, the relationship between the laser frequency and *x* can be obtained by substituting these parameters into Equation (5). Meanwhile, the transmitted intensity can be recorded (in this work 100 periods with 1000 points per period) and the FFT analysis is carried out on this signal. The Fourier coefficients at *kf* (*k* = 0, 1, 2…) frequency multiplications are extracted and used to recover the relationship between laser intensity and *x* using Equation (9). Up to now, the relationship between laser intensity and frequency can be established through the intermediate variable *x*. Then, by combining the baseline expression (Equation (13)), the absorption line profile, and the Beer–Lambert law (Equation (15)), the baseline and absorbance can be recovered simultaneously. Finally, the spectroscopic parameters [14,15] (including the collisional broadening coefficient, Dicke narrowing coefficient, line strength and so on) and the gas parameters [17,37] (such as the temperature, pressure, and concentration) can be calculated accordingly.

## 5. Conclusions

Considering the pros and cons of DAS and WMS, in this paper, a calibration-free WM-DAS method is developed based on FFT analysis to accurately recover the absorbance. This method combines the advantages of both DAS and WMS, which directly measures the absorbance with enhanced noise rejection and sensitivity. The following contents are investigated in-depth:

(1). To improve the accuracy of laser frequency and intensity descriptions, nonlinear components up to 3*ω* are considered in the description of the FM and IM response. With a scan rate of 1 kHz and a scan range of 0.866 cm^−1^, the relative best-fit residuals of FM and IM were improved to the order of 1.0 × 10^−4^.
(2). To enhance the SNR of the absorbance measurement, the characteristic spectra from FFT analysis (*kf*, *k* = 0, 1, 2, …) are extracted and used to recover the transmitted intensity. Thus, the interferences at other frequencies (electromagnetic, vibration, etc.) as well as white noise are filtered out effectively.
(3). To eliminate the uncertainty of the baseline determination, a simultaneous fitting strategy for both baseline and absorbance are performed. Since the “non-absorption” zone is not required, the laser scan range can be reduced to a great extent, which helps a lot when using high scan rate (narrow wavelength scan range) or under high-pressure condition (large spectral line collisional broadening).

In conclusion, the purposed WM-DAS method has the merits of simple operation, high-frequency calibration precision, clear baseline formula, strong anti-interference ability, high SNR, fast response, and narrow wavelength scan range. It has huge potential in scenarios such as high-accurate measurement of the spectroscopic parameter, weak absorption conditions and high-accurate gas parameter measurement in industry fields. The detailed features and applications of WM-DAS will be introduced in *Part II*.

## References

[1] Hanson R.K., Davidson D.F. (2014). Recent advances in laser absorption and shock tube methods for studies of combustion chemistry. Prog. Energ. Combust..

[2] Goldenstein C.S., Spearrin R.M., Jeffries J.B., Hanson R.K. (2017). Infrared laser-absorption sensing for combustion gases. Prog. Energ. Combust..

[3] Allen M.G. (1998). Diode laser absorption sensors for gas-dynamic and combustion flows. Meas. Sci. Technol..

[4] Deng H., Sun J., Yu B.L., Li J.S. (2015). Near infrared diode laser absorption spectroscopy of acetylene between 6523 and 6587 cm^−1^. J. Mol. Spectrosc..

[5] Cai T.D., Wang G.S., Cao Z.S., Zhang W.J., Gao X.M. (2014). Sensor for headspace pressure and H_2_O concentration measurements in closed vials by tunable diode laser absorption spectroscopy. Opt. Laser Eng..

[6] Zheng S., Liang W.K., Chu H.Q., Zhou H.C. (2020). Effect of radiation reabsorption of C_1_-C_6_ hydrocarbon flames at normal and elevated pressures. Fuel.

[7] Tabib-Azar M., Sutapun B., Petrick R., Kazemi A. (1999). Highly sensitive hydrogen sensors using palladium coated fiber optics with exposed cores and evanescent field interactions. Sensor Actuat. B-Chem..

[8] Reuter S., Sousa J.S., Stancu G.D., Hubertus Van Helden J. (2015). Review on VUV to MIR absorption spectroscopy of atmospheric pressure plasma jets. Plasma Sources Sci. Technol..

[9] Hanson R.K. (2011). Applications of quantitative laser sensors to kinetics, propulsion and practical energy systems. Proc. Combust. Inst..

[10] Stewart G., Johnstone W., Bain J., Ruxton K., Duffin K. (2011). Recovery of absolute gas absorption line shapes using tunable diode laser spectroscopy with wavelength modulation—Part I, theoretical analysis. J. Lightwave Technol..

[11] Bain J.R.P., Johnstone W., Ruxton K., Stewart G., Lengden M., Duffin K. (2011). Recovery of absolute gas absorption line shapes using tunable diode laser spectroscopy with wavelength modulation—Part II: Experimental investigation. J. Lightwave Technol..

[12] Pogány A., Klein A., Ebert V. (2015). Measurement of water vapor line strengths in the 1.4–2.7 µm range by tunable diode laser absorption spectroscopy. J. Quant. Spectrosc. Radiat..

[13] Goldenstein C.S., Hanson R.K. (2015). Diode-laser measurements of linestrength and temperature-dependent lineshape parameters for H_2_O transitions near 1.4 µm using Voigt, Rautian, Galatry, and speed-dependent Voigt profiles. J. Quant. Spectrosc. Radiat..

[14] Goldenstein C.S., Jeffries J.B., Hanson R.K. (2013). Diode laser measurements of linestrength and temperature-dependent lineshape parameters of H_2_O-, CO_2_-, and N_2_-perturbed H_2_O transitions near 2474 and 2482 nm. J. Quant. Spectrosc. Radiat..

[15] Li H., Farooq A., Jeffries J.B., Hanson R.K. (2008). Diode laser measurements of temperature-dependent collisional-narrowing and broadening parameters of Ar-perturbed H_2_O transitions at 1391.7 and 1397.8 nm. J. Quant. Spectrosc. Radiat..

[16] Liu J.T.C., Jeffries J.B., Hanson R.K. (2004). Wavelength modulation absorption spectroscopy with 2*f* detection using multiplexed diode lasers for rapid temperature measurements in gaseous flows. Appl. Phys. B-Lasers Opt..

[17] Witzel O., Klein A., Meffert C., Wagner S., Kaiser S., Schulz C., Ebert V. (2013). VCSEL-based, high-speed, in situ TDLAS for in-cylinder water vapor measurements in IC engines. Opt. Express.

[18] Ray A., Bandyopadhyay A., Ray B., Biswas D., Ghosh P.N. (2004). Line-shape study of water vapor by tunable diode laser spectrometer in the 822-832 nm wavelength region. Appl. Phys. B-Lasers Opt..

[19] Du Y.J., Peng Z.M., Ding Y.J. (2018). High-accuracy sinewave-scanned direct absorption spectroscopy. Opt. Express.

[20] Hangauer A., Chen J., Strzoda R., Ortsiefer M., Amann M. (2008). Wavelength modulation spectroscopy with a widely tunable InP-based 2.3 µm vertical-cavity surface-emitting laser. Opt. Lett..

[21] Schilt S., Thevenaz L., Robert P. (2003). Wavelength modulation spectroscopy: Combined frequency and intensity laser modulation. Appl. Opt..

[22] Li H.J., Rieker G.B., Liu X., Jeffries J.B., Hanson R.K. (2006). Extension of wavelength-modulation spectroscopy to large modulation depth for diode laser absorption measurements in high-pressure gases. Appl. Opt..

[23] Du Y.J., Lan L.J., Ding Y.J., Peng Z.M. (2017). Measurement of the absolute absorbance based on wavelength modulation spectroscopy. Appl. Phys. B-Lasers Opt..

[24] Wainner R.T., Green B.D., Allen M.G., White M.A., Stafford-Evans J., Naper R. (2002). Handheld, battery-powered near-IR TDL sensor for stand-off detection of gas and vapor plumes. Appl. Phys. B-Lasers Opt..

[25] Du Y.J., Peng Z.M., Ding Y.J. (2020). A high-accurate and universal method to characterize the relative wavelength response (RWR) in wavelength modulation spectroscopy (WMS). Opt. Express.

[26] Gustafsson J., Chekalin N., Axner O. (2003). Improved detectability of wavelength modulation diode laser absorption spectrometry applied to window-equipped graphite furnaces by 4th and 6th harmonic detection. Spectrochim. Acta B.

[27] Peng Z.M., Ding Y.J., Che L., Li X.H., Zheng K.J. (2011). Calibration-free wavelength modulated TDLAS under high absorbance conditions. Opt. Express.

[28] Goldenstein C.S., Strand C.L., Schultz I.A., Sun K., Jeffries J.B., Hanson R.K. (2014). Fitting of calibration-free scanned-wavelength-modulation spectroscopy spectra for determination of gas properties and absorption lineshapes. Appl. Opt..

[29] Sun K., Chao X., Sur R., Goldenstein C.S., Jeffries J.B., Hanson R.K. (2013). Analysis of calibration-free wavelength-scanned wavelength modulation spectroscopy for practical gas sensing using tunable diode lasers. Meas. Sci. Technol..

[30] Rieker G.B., Jeffries J.B., Hanson R.K. (2009). Calibration-free wavelength-modulation spectroscopy for measurements of gas temperature and concentration in harsh environments. Appl. Opt..

[31] Kuai S.L., Meldrum A. (2008). High-speed color-switching silicon LEDs. Adv. Mater..

[32] McGettrick A.J., Duffin K., Johnstone W., Stewart G., Moodie D.G. (2008). Tunable diode laser spectroscopy with wavelength modulation: A phasor decomposition method for calibration-free measurements of gas concentration and pressure. J. Lightwave Technol..

[33] Chen Y.X., Xu D.L., Xu K.K., Zhang N., Liu S.Y., Zhao J.M., Luo Q., Snyman L.W., Swart J.W. (2018). Optoelectronic properties analysis of silicon light-emitting diode monolithically integrated in standard CMOS IC. Chin. Phys. B.

[34] Peng Z.M., Ding Y.J., Che L., Yang Q.S. (2012). Odd harmonics with wavelength modulation spectroscopy for recovering gas absorbance shape. Opt. Express.

[35] Peng Z.M., Ding Y.J., Jia J.W., Lan L.J., Du Y.J., Li Z. (2013). First harmonic with wavelength modulation spectroscopy to measure integrated absorbance under low absorption. Opt. Express.

[36] Lan L.J., Ding Y.J., Peng Z.M., Du Y.J., Liu Y.F., Li Z. (2014). Multi-harmonic measurements of line shape under low absorption conditions. Appl. Phys. B-Lasers Opt..

[37] Du Y.J., Peng Z.M., Ding Y.J. (2018). Wavelength modulation spectroscopy for recovering absolute absorbance. Opt. Express.

[38] Gordon I.E., Rothman L.S., Hill C., Kochanov R.V., Tan Y., Bernath P.F., Birk M., Boudon V., Campargue A., Chance K.V. (2017). The HITRAN2016 molecular spectroscopic database. J. Quant. Spectrosc. Radiat..

[39] VMalathy Devi DChris Benner Smith M.A.H., Mantz A.W., Sung K., Brown L.R. (2012). A Predoi-Cross. Spectral line parameters including temperature dependences of self- and air-broadening in the 2←0 band of CO at 2.3 µm. J. Quant. Spectrosc. Radiat..

